# Healthcare workers’ perspective about barriers and facilitators to pediatric HIV status disclosure in eastern Uganda using capability opportunity and motivation of behavior change model

**DOI:** 10.1371/journal.pgph.0004662

**Published:** 2025-05-29

**Authors:** Joseph Kirabira, Godfrey Zari Rukundo, Brian C. Zanoni, Celestino Obua, Edith Wakida, Christine Etoko Atala, Naume Etoko Akello, Keng-Yen Huang, Scholastic Ashaba

**Affiliations:** 1 Department of Psychiatry, Busitema University, Mbale, Uganda; 2 Department of Psychiatry, Mbarara University of Science and Technology, Mbarara, Uganda; 3 Departments of Medicine and Pediatric Infectious Diseases, Emory University School of Medicine, Atlanta, Georgia, United States of America; 4 Office of Vice Chancellor, Mbarara University of Science and Technology, Mbarara, Uganda; 5 Department of Anaesthesia, Mbarara University of Science and Technology, Mbarara, Uganda; 6 Department of Psychology, Makerere University, Kampala, Uganda; 7 School of Medicine, New York University, New York, United States of America; University of Embu, KENYA

## Abstract

HIV status disclosure by caregivers to children and adolescents living with HIV (CALH) remains a public health concern in countries with a high burden of HIV despite guidelines for healthcare workers (HCWs) to facilitate the process. This study explored barriers and facilitators to HIV disclosure at two referral hospitals in eastern Uganda focusing on the utilization of existing guidelines. In-depth qualitative interviews were conducted among all HCWs involved in the management of CALH at three pediatric HIV clinics. Research assistants collected data using a semi-structured interview guide designed based on the Capability, Opportunity, and Motivation of Behavior change (COM-B) Model. The audio-recorded interviews were transcribed verbatim, analyzed thematically, and categorized based on the COM-B and social-ecological models using the inductive content approach. Sixteen in-depth interviews were conducted among HCWs, including both males and females in equal numbers. The barriers to disclosure involved all five levels of the social-ecological model, while facilitators were at only three levels (individual, interpersonal, and institutional levels). Regarding the capability of HCWs to support disclosure, limited training affected their psychological (knowledge) and physical ability (skills), while awareness of responsibilities enhanced psychological ability. For opportunity, an unstable home environment, limited access to guidelines, and HIV-related stigma were barriers in physical and social environments, while peer support, teamwork, and orphanhood status were facilitators in the social environment. Limited health funding and lack of preparatory procedures affected reflective motivation, while delayed disclosure affected automatic motivation. Conversely, emotional reward and monitoring, checklists, and supervision enhanced the automatic motivation of HCWs toward disclosure. The findings highlighted several potentially modifiable factors that need to be addressed or reinforced to improve HIV disclosure and utilization of existing guidelines. These findings are key in informing stakeholders regarding the development of implementation strategies for improving pediatric HIV disclosure and utilization of existing guidelines in Uganda.

## Author's summary

HIV status disclosure to children and adolescents is important for good treatment outcomes but remains a public health challenge in many countries grappling with a high burden of HIV infection regardless of the existing guidelines. This study was conducted among sixteen purposively selected HCWs to explore barriers and facilitators to HIV status disclosure to children and adolescents living with HIV in eastern Uganda. Qualitative interviews were conducted using a guide developed based on the Capability, Opportunity, and Motivation model of behavior change. Findings indicated that limited training affected both the psychological and physical ability of HCWs to support HIV disclosure, while awareness of responsibilities was a facilitator. An unstable home environment, limited access to guidelines, and HIV-related stigma were physical and social environment barriers, while peer support, teamwork among HCWs, and orphanhood status were social facilitators. Limited health funding, lack of preparatory procedure, and delayed disclosure affected reflective and automatic motivation, while emotional reward to HCWs and monitoring, checklists, and supervision were automatic motivators.

There is a need to design interventions that address these barriers and reinforce the facilitators to enhance the capability and motivation of HCWs to support disclosure and to improve the social and physical environments for disclosure.

## Background

By the end of 2023, about 120,000 children aged below 15 years were newly infected with HIV, and the majority are in low-income countries [1]. In seven selected sub-Saharan Africa, 450,000 children aged between 0–14 years were living with HIV by 2017 with a weighted prevalence of 0.7-1.1% [2,3]. By 2023, 72,000 children below 14 years were living with HIV, with about 3200 AIDS-related deaths in Uganda [4]. Most children acquire HIV vertically either in utero, during childbirth, or through breastfeeding [5–7]. During the early years of life, many children receive HIV care without knowing their HIV status due to stigmatization, fear of disclosing the mother’s status, lack of knowledge about the disclosure process, fear of children’s reaction, or considering that the child is still too young to understand [8–12]. The World Health Organization (WHO) and Uganda Ministry of Health recommend that caregivers of children and adolescents living with HIV (CALH) should be supported to disclose the HIV status of their children by trained healthcare workers during the pre-disclosure, disclosure, and post-disclosure stages [13,14]. Elizabeth Glaser Pediatric AIDS Foundation also emphasizes that children and their parents should undergo intensive stepwise pre-and post-disclosure counseling and mental health assessment by healthcare workers (HCWs) [15]. Regardless of the above recommendations, pediatric HIV status disclosure remains a challenge in most low- and middle-income countries (LMICs), pointing to possible challenges at the level of healthcare workers. Unfortunately, most existing literature has focused on exploring barriers to HIV status disclosure from the perspectives of caregivers and adolescents rather than HCWs, who are the main facilitators of the disclosure process [16,17]. Exploring the perspectives of healthcare workers concerning HIV disclosure can provide critical information relevant to formulating interventions based on their interaction with CALH, their caregivers, and health guidelines or policymakers. Additionally, most of the existing studies have not applied standard implementation research models or frameworks in exploring these existing barriers and facilitators to pediatric HIV status disclosure [9,18]. The capability Opportunity and Motivation of Behavior change (COM-B) model is a determinant framework that postulates that a person’s engagement in specific health behavior (in this case, HIV disclosure) is determined by their physical and psychological ability (capability), physical and social environments (opportunity) and reflective and automatic motivation [19]. Unlike other determinant models or frameworks, this model provides a simplified systematic approach to assessing determinants (both barriers and facilitators) of health behavior that can be broadly applied in various contexts [20]. Uniquely, information obtained using the COM-B model can be fed into the behavioral change wheel to guide the formulation of appropriate interventions to address the identified barriers [19]. Therefore, this study aimed to explore the HCWs’ perspectives regarding barriers and facilitators to pediatric HIV status disclosure at three regional referral HIV Clinics in Eastern Uganda using the Capability, Opportunity, and Motivation model of Behavioral Change [19]. The study considered the utilization of existing Uganda Ministry of Health or WHO disclosure guidelines by HCWs to facilitate the disclosure process.

## Methods and materials

### Ethics statement

This study was approved by the Mbarara University of Science and Technology Research Ethics Committee and Uganda National Council of Science and Technology, approval numbers MUST-2022–704 and HS4017ES respectively. All participants provided written informed consent before participating in the study.

### Design and study setting

A qualitative study was conducted among healthcare workers who were involved in the management of children and adolescents living with HIV (CALH) attending three clinics located at two regional referral hospitals in Eastern Uganda. The three clinics were the Infectious Disease Clinic (IDC) and The AIDS Support Organization (TASO) clinic in Mbale Regional Referral Hospital (MRRH), as well as the Jinja Regional Referral Hospital (JRRH) pediatric HIV clinic. MRRH and JRRH are the largest hospitals in the region and provide specialized and general medical services to people from more than 10 districts with a catchment population of about 4.15 million people [21]. The pediatric HIV clinics are part of the respective pediatric departments and mainly provide HIV/AIDS care on an outpatient basis, while any admissions are referred to the general pediatric wards. IDC, TASO (at MRRH), and JRRH offer HIV/AIDS services to about 1050, 350, and 500 CALH aged 0–18 years annually, respectively with the average age of children for HIV status disclosure being 10 years. IDC and JRRH pediatric HIV clinics are run by the public health service system and financed mainly by the government, while the TASO clinic is privately run by TASO, a non-government organization. Both hospitals (MRRH and JRRH) serve as teaching sites for various training institutions, whereby MRRH is a teaching site for Busitema University Faculty of Health Sciences while JRRH is a teaching hospital for Kampala International University. Pediatric HIV care services are usually offered by a team of clinicians ranging from pediatricians, postgraduate students from respective training institutions, general medical doctors, clinical officers, nurses, counselors, and social workers, among others.

### Study population

The study targeted all healthcare workers involved in the day-to-day management of CALH at any of the above three clinics. We included all healthcare workers formally employed by the clinics for assessment and management of HIV/AIDS-related problems among children and adolescents on either outpatient or community outreach basis since the clinics do not run inpatient services. These included medical officers, clinical officers, nurses, counselors, medical social workers, and peer supporter leaders. All healthcare workers from other clinics in the hospital who only review CALH upon special consultations were excluded from the study.

### Participant recruitment, sampling, and data collection

Potential participants were identified during routine working days and hours as they attended the clinic for review by trained research assistants (NEA – a female psychologist, and RK – a male pre-intern doctor). Both research assistants had prior experience in collecting such data and received additional training before conducting interviews during this study. They were initially involved in working with some of the participants during the collection of quantitative data from CALH who attend the same clinics. Data collection started on 01/August/2023 and ended on 26/January 2024. The RAs approached potential participants face-to-face, explained the purpose of the study, and requested their consent to participate in the study. Those willing to participate would choose their most convenient time to have the interview to minimize the disruption to their clinical work and to provide ample time for the interview.

Participants were selected purposively to involve only those HCWs involved in the management of CALH, and they were recruited consecutively. All eligible staff at each clinic were approached by trained research assistants for participation, and only two declined. One nurse was uncomfortable with being recorded, while one social worker cited that her contract barred her from participating in such studies. The sample size was determined by the availability of eligible participants rather than data saturation. Data were collected by the research assistants through conducting face-to-face, in-depth interviews using a piloted interview guide (S2 Table). The guide was adapted from a study by Wakida et al., which was developed using the Capability, Opportunity, and Motivation of Behavior (COM-B) model in southwestern Uganda to explore barriers and facilitators to the integration of mental health services into primary health care [22]. This model, developed by Michie et al. in 2011, has three main domains, which include Capability, Opportunity, and Motivation. [19] It was based on the understanding that a person is required to have the capability, opportunity, and motivation to engage in a certain behavior, which in this case is supporting HIV status disclosure [23]. Therefore, barriers and facilitators to pediatric HIV status disclosure were explored using this model from the perspective of HCWs. Capability is the psychological or physical ability of a person to engage in a behavior. Sample interview questions for psychological and physical abilities included “*Would you remember to follow the guidelines when facilitating HIV status disclosure?*” and “*How do you disclose HIV status to children?”* respectively. Opportunity refers to physical or social environments in which behavior is conducted, and sample interview questions included *“To what extent does the surrounding environment facilitate or hinder HIV status disclosure?”* (for the physical environment) and *“To what extent do social influences facilitate or hinder facilitation of HIV status disclosure?*” (for the social environment). Motivation focuses on a person’s reflective or automatic mechanisms to carry out behavior, and sample questions included *“Do you think you need to use the guideline during the disclosure process? Why?”* (for reflective motivation) and *“Does facilitating the HIV status disclosure process cause some emotions in you?”* (for automatic motivation). Overall, the interview guide included questions that assess barriers to disclosure in general as well as those that mainly focused on the use of MoH or WHO disclosure guidelines. The COM-B model can be used to design appropriate interventions based on the identified barriers and facilitators as guided by the Behavior Change Wheel [19]. All interviews were audio recorded, and additional field notes were taken by the research assistants. Each interview lasted between 30–40 minutes, and participants received refreshments during interviews and modest compensation for their time at the end of the interview.

Privacy was guaranteed by ensuring that all interviews or assessments were conducted within a safe, secure, and private place with only one participant at a time to ensure that participants freely express themselves with no interference from colleagues and no repeat interviews were conducted.

### Data analysis

Each participant was assigned a unique identification code, and data were analyzed thematically. The audio recordings were transcribed verbatim by NEA (a research team member) and were initially read for familiarization. Later, the transcripts were coded using Atlas.ti version 23.4 software to form themes using inductive content analysis under three phases: open coding, creating categories, and abstraction. Coding was initially done independently by two researchers experienced in HIV-related qualitative research. (PN – who is not part of the authorship team, and JK – who is one of the authors). Any discrepancies were resolved by involving a third researcher with more experience (mentor/supervisor) to reach a consensus (SA – one of the co-authors). The themes were harmonized and categorized by considering manifest and latent content. The themes were further organized using the social-ecological model. Results were reported following the COnsolidated criteria for REporting Qualitative research (COREQ) checklist (S1 Table) [24].

### Ethical considerations

The study was approved by the Mbarara University of Science and Technology Research Ethics Committee (approval number MUST-2022–704) and registered with the Uganda National Council of Science and Technology (number HS4017ES). All participants provided written informed consent before participating in the study. All data collection tools and consent forms were in English, the Ugandan official language. Privacy was ensured during the entire data collection process by conducting interviews with one person at a time from a private and secure room or space within the respective health facility. Each participant was assigned a unique identifier to ensure anonymity, and all collected information (especially recordings) was stored in a locker only accessible by the principal investigator. During data transcription, analysis, and manuscript writing, all participants’ identifying information was removed to further ensure confidentiality.

## Results

Sixteen (16) participants (equal males and females) were recruited in the study, with the majority (n = 8) from the IDC (see Table 1), and only two eligible participants declined to participate in the study.

**Table 1:** Characteristics of HCWs that were interviewed (N = 16).

Participant characteristic	Frequency (n)	Percentage (%)
**Job description**		
Counselors	3	18.8
Nurse	2	12.5
Clinical officer	3	18.8
Medical officer	1	6.2
Medical social worker	3	18.8
Young children and adolescent peer supporters	4	25.0
**Sex**		
Males	8	50.0
Females	8	50.0
**Study site**		
TASO Mbale	5	31.2
IDC - Mbale	8	50.0
Jinja Pediatric HIV clinic	3	18.8

### Barriers and facilitators to pediatric HIV status disclosure and utilization of existing disclosure guidelines

Table 2 summarizes these barriers and facilitators according to the different constructs of the capability, opportunity, and motivation domains of the COM-B model. The most common barriers to HIV disclosure reported were HIV-related stigma, followed by a lack of pediatric HIV disclosure training among healthcare workers and limited access to disclosure guidelines. On the other hand, peer support among CALH, their caregivers, and HCWs was the most common facilitator of HIV disclosure.

**Table 2:** Barriers and facilitators of pediatric HIV status disclosure focusing on MoH and WHO disclosure guidelines according to the COM-B model.

DOMAIN	CONSTRUCTS	BARRIERS	FACILITATORS
**CAPABILITY**	Psychological ability	Limited training in pediatric disclosure (lack of knowledge)	HCW’s awareness of responsibilities
	Physical ability	Limited training in pediatric disclosure (lack of skills)	
**OPPORTUNITY**	Physical environment	Unstable home environment	
		Limited access to guidelines	
	Social environment	Enacted, Internalized, and anticipated stigma	Peer support
			Teamwork among healthcare workers
			Orphanhood status
**MOTIVATION**	Reflective mechanisms	Limited health funding	
		Lack of preparatory procedures	
	Automatic mechanisms	Delayed disclosure	Emotional reward to HCWs
			Checklist, monitoring, supervision

Using the social-ecological model, the themes related to barriers and facilitators were further organized under five levels, which included individual, interpersonal/relationships, institutional, community, and policy levels, as indicated in S1 Fig. Below is a detailed account of the various barriers and facilitators to HIV disclosure and utilization of guidelines by HCWs.

### Individual level

#### - Internalized and anticipated stigma.

Stigma experienced by caregivers of CALH was reported as a common social barrier to HIV disclosure. HCWs reported that many caregivers, especially biological parents, experience internalized stigma where they reported feelings of unworthiness, guilt, shame, and self-blame for having infected their children with HIV. This made it very difficult for them to inform their children about their status as they thought that they would be blamed by their children. Because of this stigma, some caregivers reportedly failed to disclose to their children due to the fear to face anger, violence sadness, and suicidal behavior from their children

HCW3A/SOCIAL WORKER**: “***There’s a home I went to when a mother came here and invited me. She and her husband were going to disclose to the children. I went and reached; the woman started crying. I asked what was wrong; she said, “Yesterday, the child wanted to kill me. She is blaming me for infecting her with HIV; I did not intend to infect the child. I’m just a woman who was abandoned by her husband and by breastfeeding the child, although they told me I had nothing to eat. The husband had already gone, so I thought that by breastfeeding her for up to one and a half, I was doing something. Even giving birth, I had no health worker who was by my side. I was alone with my mother, and I think something somewhere went wrong, so I’m praying that you somehow forgive me”. So, you know she was down and in a bad situation.*

On the other hand, **orphanhood status** was reported to be a social facilitator of HIV status disclosure since the death of biological parents eliminates the mark of stigmatization and the risk of disclosing the parents’ HIV status. Hence, the rest of the relatives can easily inform the child about the illness, sometimes relating it to their parent’s death since the community presumably already knows. Additionally, family settings with no adult caregivers encourage HCWs to ensure that the child is informed of their status as early as possible.

*HCW11/ SOCIAL WORKER. “Mainly, most of the fears are around, ‘how am I going to tell my child that I’m responsible for their HIV infection?’ So, the majority would prefer not to disclose, especially if it’s a mother taking care of her biological children. But if the mother who passed on, and this adolescent has grown up in the hands of another caretaker, it is a little easier for that caretaker to disclose.”*

*HCW3B/SOCIAL WORKER: “…there’s a child-headed family, of course, where there’s nobody else. No adult or caregiver. So, you are forced to disclose to such a child.”*

#### - Delayed disclosure.

HCWs reported that when children grow up to late childhood, it becomes difficult but more urgent to disclose as such children tend to create pressure on the caregivers especially parents, and HCWs, to inform them of their illness. Delayed disclosure automatically motivates HCWs to facilitate disclosure to correct the anomaly, prevent psychosocial complications, and improve treatment outcomes. However, many reported that under such circumstances, it is difficult to follow the standard guidelines during the disclosure process.

*HCW7/NURSE: “The child was putting his mother on tension. It took us time, but we finally settled it. We talked to the mother and son and left when they were very okay otherwise, it was an issue. Why? It was because of non-disclosure. Because the child had gone 18 years without being disclosed to and he disclosed to himself.”*

*HCW4/NURSE* ***: “*** *In areas where disclosure is not done early the outcome remains a challenge, the children will remain non-suppressed or have socio-economic challenges.*

Whereas guidelines recommend disclosing according to a child’s developmental abilities, HCWs noted that it is not easy or very clear how to assess and decide about a child’s maturity or readiness for disclosure. This is because some “young” children may be very inquisitive and seem to understand the HIV concepts while “older” ones may not, hence delayed disclosure in some children.

*HCW8/CLINICIAN* ***: “*** *There are standard guidelines, though sometimes we may not be able to follow them to dot. Disclosure is a process that starts at a certain age, but bearing in mind that children’s development and growth also differ. So there are those whereby you are like this one should be disclosed to even if they are young at least they can understand what and why they are doing this.”*

*HCW2/CLINICIAN: “I’ve seen a child in p.2 who chooses to know the reason why. So, the parent comes to you saying the child wants to know why. Guidelines are good, but there are some exceptions where you see that there are some things that must be a blended truth to an inquisitive child, and maybe the earlier the child knows, the better.”*

Other times, it is the anticipation of negative emotional reactions by HCWs and caregivers from children that deters them, hence delaying disclosure.

*YAPS2: “So, if you tell the child that you know why they are taking medicine every day or disclose, the child can end up even abusing you, misbehaving, or acting weirdly. Others can bring out their anger and cry for a long time. They can make you scared that they are going to do something bad to themselves. And you feel bad and wish you didn’t disclose.”*

#### - Emotional reward to HCWs.

Some HCWs reported that positive emotional reactions expressed by children or their caregivers, like happiness, encourage them to facilitate the disclosure process as they elicit feelings of being appreciated for accomplishing such a difficult task. Additionally, appreciation from their employers or witnessing the success of some children whom they supported through disclosure were also reported to elicit similar feelings. Hence, these emotional reactions act as automatic motivators for HCWs to continue facilitating the disclosure process regardless of the other challenges faced.

*HCW5/NURSE* ***: “… t*** *here is also that one where the child can be happy and thank you for telling them. Those are children who understand. So that is the benefit you get from doing this. The other benefit you get is appreciation by parents.”*

*HCW3B/SOCIAL WORKER: “My motivation is appreciation. Sometimes, I do the work, and my bosses say thank you, and yet the payment and the resources are not big..”*

#### - HCW’s awareness of responsibilities.

HCWs who viewed disclosure as being part of their job description and responsibility were more likely to support disclosure and try to follow the guidelines as opposed to those who thought that it was not part of their work or responsibility. This enhanced the psychological ability of such HCWs (the former) by being more willing to learn about disclosure and related guidelines and remember to facilitate the process whenever possible.

*HCW10/CLINICIAN* ***: “*** *Disclosure is a part of my role because as one of the frontline health workers, one of the persons that interfaces with the clients and one of the persons to whom these caregivers or clients will disclose what they are going through, I’m expected to know how to go about the disclosure in this state. So, I have a big role to play in the disclosure process.”*

*HCW8/CLINICIAN* ***: “*** *No disclosure is not part of my job description, though you will realize that when we are in the clinic, we have a kind of role description. Issues around disclosure are more psychosocial. Now, sometimes, if you are not this kind of service provider who can say no, let me also try to understand the psychosocial part of this child, there you will be fixated on your medical bit of it, trying to do investigations, trying to discover whether this child is swallowing his medicine well.”*

### Interpersonal level

#### - Anticipated stigma.

This was a very important social barrier to disclosing HIV status to children by caregivers who have kept their status undisclosed to their partners or other family members. This is because such partners think that disclosing to children may cause accidental disclosure to the partner or other people closely related to the caregiver. This stigma is worsened by the anticipated reactions or maltreatment from such significant people around them, which may disrupt such strong relationships, hence preferring not to disclose to the child to avoid negative consequences. Consequently, such fears lead to caregivers being in denial or having negative attitudes towards HCWs who try to disclose to their children.

*HCW3B/SOCIAL WORKER***:** *Yes. Sometimes, there are those clients where the mother and child are positive, and the mother has not disclosed this to the family. They’ve not told the siblings and other people, so they cannot tell the child. They take the medicine secretly.*

*YAPS4***:** *Disclosure is sometimes difficult, and you can even be arrested for it. We have some people who don’t want totally to hear about it being told to their children because they may get where they are discordant. So, when I speak, the father may not know that the mother is positive. So, the child can go and ask Daddy why this and this, which will bring conflict.*

#### - Peer support.

HCWs reported that peer support is a very strong facilitator for HIV status disclosure to CALH as it provides a favorable social environment not only for CALH but also for their caregivers and HCWs. Peer support was reported at three different levels, which are child to child, caregiver to caregiver, and HCW to HCW. At all levels, there is sharing of experiences and ideas related to disclosure between peers and sometimes follow-up, hence making it easier for each other. Child-to-child peer support is mainly provided by Young children and Adolescent Peer Supporters (YAPS) who exist as part of the support staff in the current healthcare system.

*HCW6/COUNSELOR: “During follow-up, I can say most of these clients are attached to the expert clients, what I call the YAPS, so they are always in touch at least once every month, that is, if the adolescent has been taking good treatment. If there are cases of concern, they can forward them to us, and as the counselors, we engage them. But these children are being monitored by the YAPS, who see their adherence and overall attendance in the facility.”*

Caregiver-to-caregiver support is mainly provided by those caregivers who have successfully disclosed to their children or existing caregiver peer supporters like linkage facilitators or expert clients. This can be done during informal interaction within or outside the health facilities or during formal caregiver meetings organized by HCWs.

*HCW6/COUNSELOR: “Yes, they normally interact when they have come for their clinic day, so I believe during their interaction, they normally share experiences. For instance, a caregiver might be afraid to tell their 13-year-old child, and they’ll say my child wants to know why they take medicine. Then another caregiver will say tell him this, for me, this is how I handled mine, and we are now okay. So, through sharing of experiences, I believe the caregivers can influence their colleagues to disclose.”*

*HCW11/ SOCIAL WORKER: “In our caregivers’ meetings, we call out those who have done it well to share their experiences and how they handled the challenges. So even those who are scared pick up slowly.”*

HCWs reported that those who routinely talk about or conduct disclosure act as a good influence on others, hence motivating them to also get involved in facilitating caregivers to disclose. Also, the availability of technical people and supervisors encourages others to participate, knowing that they will be supported in case of any challenges during the disclosure process. Such a supportive social environment boosts confidence and encourages HCWs to engage in facilitating HIV disclosure to children and adolescents seeking care at their respective facilities.

*HCW6/COUNSELOR: “Then on the side of healthcare workers, the influence of disclosure may be that sometimes, as we give health education in groups, you find that a certain health worker is talking more about HIV disclosure during the talks, so others can learn from him/her.”*

*HCW6/COUNSELOR: “The hospital environment facilitates the process because we have technical people, counselors who are trained.”*

Additionally, some HCWs reported that sometimes, “supported disclosure” is conducted whereby several relatives, friends, community health workers, and HCWs meet and disclose to a child with consent from the primary caregiver. This process is mainly driven by community health workers like expert clients, linkage facilitators, mentor mothers, and village health team members by identifying children who have not been disclosed to and hence working with the HCWs to ensure that disclosure is conducted.

*HCW3B/SOCIAL WORKER: “So, the parent can say I’ve given you that child, you go in a room and talk. Sometimes they come as a family, father, mother, and other siblings, with peers and I, we disclose to the child.”* ***“****Yes, it’s a group disclosure - a group of about seven.”*

*HCW11/ SOCIAL WORKER* ***: “*** *All the PLHIV have community health workers attached to them. Categories of community health workers are the YAPS, mentor mothers, and linkage facilitators. So, all active clients in care have someone attached to them. So, when we notice it’s child A having a problem with support or disclosure, we encourage their attached supporter to follow up on them. So, they will be like middlemen between families and facilities. They will give us the information and we can give the support. We cannot do it on the clinic day because it may be overwhelming.”*

#### - Teamwork.

This was also mentioned as one of the facilitators in relation to the social environment of HCWs. Some HCWs highlighted that working as a team whereby everyone gets involved in disclosure to do their part well encourages them and improves the disclosure process and experience.

*HCW5/NURSE: “Teamwork can improve disclosure. When you work in a team you love your job. Where there is no teamwork you start finger-pointing that so and so is supposed to do this.”*

*HCW9/CLINICIAN: “And the people you are working with; they are a very good motivator. If they like and they embrace whatever you introduce to them, that would be good for disclosing to our children.”*

### Institutional level

#### Lack of disclosure training for HCWs.

Most HCWs at the HIV clinics reported not having received any disclosure training while those who were trained also reported a need for refresher training to cope with the changing standards of practice. This was reflected by the inadequate knowledge about the pediatric HIV status disclosure process or guidelines. Lack of disclosure training affected the psychological and physical abilities of HCWs due to a lack of adequate knowledge and skills respectively.

*HCW11/ SOCIAL WORKER* ***: “*** *It is a hard thing, and we realize that some healthcare workers have not been trained. The majority of our healthcare workers are not trained, even when you sit in their disclosure sessions, it is trouble. So I’d recommend that we have training, especially for those who work with adolescents to have more information on how to go about the disclosure process because whatever happens in the disclosure process will either negatively or positively affect this young person for the rest of their lives.”*

*HCW2/CLINICIAN: “If you don’t know what to say, at what time, and how to say it, it becomes a challenge. Children ask a lot of questions, so if I don’t know how and what to answer them, it becomes a problem to disclose. But if I’m empowered, if I know what to say at that time, and how to say it to a child, that one really facilitates and makes it a bit easy for me to do it.”*

#### - Lack of preparatory procedure.

Some HCWs reported that the lack of proper procedures and steps to follow in preparation for disclosure at their clinics such as accessing a child’s medical records and documenting his/her disclosure status demotivates them from facilitating the disclosure process. Hence HCWs believe that the HIV clinics need to put in place pre-disclosure preparatory procedures with proper planning to improve the disclosure process and as a result, motivate HCWs.

*HCW3B/SOCIAL WORKER: “Purposely for disclosure? No, there are no steps that can prepare the facilitation of disclosure because normally they say we integrate. When we are going for a refill, follow-up of a missed appointment, when you have a disclosure, you integrate.”*

*YAPS2: “I always had that in mind that especially when we are going to do disclosure, it’s good for the organization to create a list of children that don’t know why they are taking medicine. We can make for them a clinic of 15-20 so that we can plan and facilitate them and in this, we are looking for a point of them knowing why they’re taking their medication.”*

#### - Monitoring, checklists, and supervision.

Some HCWs reported that they were motivated to facilitate disclosure by knowing that their work is being monitored or supervised by their employers through directly observed disclosure sessions, reviewing some specific clinic records and audit tools. This would make them engage in disclosure and related activities since it was key to their performance.

*HCW8/CLINICIAN: “So the psychosocial services are well supervised. We also have what we call sitting sessions, where somebody can choose maybe once a month to look at and try to monitor a specific person [a HCW] by sitting in that session. But sometimes we also do what we call file reviews then we can date back that person and see.”*

Additionally, HCWs reported several routine findings, clinical considerations, or activities that regularly remind them of the need and automatically motivate them to support caregivers to disclose to their children. These included the clinical status of children such as those who are viral non-suppressing, having comorbid medical illnesses, and poor adherence to treatment. Also, children at transitional stages such as childhood to adolescence or adolescence to adulthood were always considered critical for disclosure as these would undergo care-related changes like changes in clinics.

*HCW11/ SOCIAL WORKER: “… as a clinic, we are supposed to schedule 10-18 years for the adolescent clinic. But before we schedule them, we need to make sure that they have been disclosed to so when they clock 10 years, we usually ask the parents or caretakers, has your child been disclosed to? If they say no, then we start discussing with him or her how they can go about it.”*

*HCW3A/SOCIAL WORKER: “We have the audit tool which we use. It is because we collect the primary data, it is entered into the system. So, our M&E can generate the data. Has the person been disclosed to? Yes, or no so we know which children have been disclosed to and this ensures that disclosure is done.”*

### Community level

#### - Enacted Stigma of HIV.

Like at individual and interpersonal levels, enacted HIV-related was highlighted as a major social barrier to disclosure experienced by CALH and their caregivers both in the community (and schools for CALH) and hospital settings. This is because of people’s negative perceptions and misconceptions towards HIV/AIDS which make caregivers feel stigmatized hence fearing that when they tell the children, other people will know which will worsen the discrimination towards both child and caregiver*.*

*HCW9/CLINICIAN: “When it comes to hindering, its stigma. When someone is growing up in school and our community no one says anything good about HIV. So, in such instances where they talk bad things about HIV, you can imagine when you tell them that you’ve HIV and you are going to take these medicines. So, the community itself has not done good to us.”*

*HCW3A/SOCIAL WORKER* ***: “*** *One, there’s rejection on the side of the child. Some children are rejected by caretakers. So, when you disclose to help the child, you are causing rejection. Sometimes people in the community can also reject the caretaker. That for you only give birth to only positive children. So, disclosure is good, but we also see the negative side.”*

#### - Unstable child’s home environment.

Children from homes characterized by changing caregivers, separated or stepparents and those that cannot easily be accessed (such as grandparents or those with severe mental illness or disabilities) were reportedly having challenges with disclosure. This was because some caregivers such as sex workers never have enough time for the children to disclose to them while other children have multiple caregivers who change from time to time affecting the education/training aspects of the disclosure process. As a result, this presents as a disclosure barrier related to the physical environment in which the child or adolescent lives.

*HCW4/NURSE: “And now to children, when a child is born to a mother who is a sex worker, has no time to tell the child that you have a,b,c,d. Then children are cared for by many people. For example, when the mother delivered and died and the aunt took over. The aunt who took over does not provide care thus the next person takes over.”*

*HCW6/COUNSELOR***:** *The challenges are that some adolescents have multiple caregivers, for instance, today he comes with the mother, you start disclosure, talk about it, encourage the mother to start the process, and counseling of disclosure. This mother accepts that she will talk to him and on the next visit we shall be ready. but the next visit he comes with a grandparent and the grandparent says first wait so it delays. Another time maybe the uncle brings this child. So you find maybe the child is having so many caregivers who normally bring him/her to the clinic, it lengthens the process.*

### Policy level

#### - Limited access to guidelines.

This was another barrier related to the physical environment under which HCWs facilitate the disclosure process. Most of the HCWs report having no or limited access to pediatric HIV status disclosure guidelines issued by local authorities or the World Health Organization which negatively impacts their ability to facilitate disclosure. Due to this unavailability, some resorted to using other guidelines (such as adherence counseling guidelines) or job aids that are not primarily designed for pediatric HIV status disclosure while others disclose or facilitate the disclosure process without following any guideline that predisposes children to unsafe disclosure.

*HCW3B/SOCIAL WORKER* ***: “*** *Sometimes these guidelines are not in the vicinity, especially to us who are on the ground. Though they might be in those [higher] offices, they’re not here for us. So, we need to access them.”*

*HCW10/CLINICIAN* ***: “*** *I would ideally remember to follow the guidelines during the disclosure process, to follow the steps. And as well be flexible according to the situation. Unfortunately, these are guidelines that are not displayed, they are not readily available to everyone. You know because the disclosure process is one that you will not predict, that it’s going to happen at this time, or you will need to do it this time. I feel these guidelines should be either pinned in the form of SOPs or having a desktop job aid for a health worker to refer to at the time when this need arises”.*

Additionally, those who could access the guidelines reported that they are lengthy and time-consuming hence being difficult to apply given time constraints in their clinics. Consequently, they advocated for the provision of summarized versions of the guidelines which can be easily applied in real clinical settings.

*HCW10/CLINICIAN* ***: “*** *Using the guidelines should be easy. One that does not take a lot of time because we have health workers with quite a lot to do during the day. I look at the ideal guideline as one that is as short as possible but elaborate enough to guide the health worker in that process”.*

#### - Inadequate health funding.

HCWs reported many challenges for HIV status disclosure which were directly related to the inadequate health financing which makes the environment in health facilities unfavorable for facilitating disclosure. These challenges included inadequate staffing level which leads to work overload by the few available staff and posing time constraints due to conflicting schedules. Consequently, this demotivates the few staff making them unable to support disclosure due to competing interests.

*HCW10/CLINICIAN* ***: “*** *Also, time is a factor, is something that some of us may not have given the number of patients to attend to. For example, the clients you have before you have to have the disclosure process started. But then you have a long line of clients to attend to. And this there are two outcomes, you may not ably or not satisfactorily support the disclosure process or not do it at all because the priorities here are competing.”*

However, to navigate the above, some HCWs reported that pre-disclosure planning helps them to prepare well and give enough time to the clients at their convenience. Additionally, this allows them to organize all the necessary documents including guidelines to be followed during the process.

*HCW4/NURSE: “You arrange and give the person enough time both for allowing anxiety, for questions for guidance, and for allowing the client to decide. And if it’s a child you have sessions, one session for the parents, one parent or both, later on you find out the understanding of each other”*

Also, HCWs reported that clinics that lacked private spaces where HCWs could discuss disclosure-related issues with caregivers and/or children were not conducive to facilitating the disclosure process as this affects the ability to share sensitive health information in fear that other people will get to know. This endangers patients’ privacy and confidentiality of their information hence demotivating HCWs from engaging in disclosure activities.

*HCW6/COUNSELOR: “As an adolescent clinic, we lack privacy, the space, sometimes you find there are other people, and the adolescents are not always comfortable with adults. So, at times you don’t find that favorable space or place to offer the disclosure. Because someone you disclose to, you don’t anticipate how this person might react. This person might cry, and everyone is looking at them so sometimes it hinders.”*

Finally, HCWs reported that limited facilitation in terms of children’s welfare, stationery, and other aids to carry out disclosure-related activities in clinic settings is a barrier to disclosure. Also, limited facilitation in terms of transport, and airtime for communication, among others, to follow up and conduct disclosure activities in the community was cited as a barrier to HIV status disclosure as it demotivates HCWs from supporting the process/activities.

*HCW4/NURSE: “There are some facilitations which are not done, like here for example where you have children who need disclosure, and you need like porridge for them, it remains a problem. Having objective charts, stationary materials, dolls, etc. also contribute to communication.”*

*HCW1/COUNSELOR* ***: “*** *Sometimes we need facilitation of follow up because we need to follow up these clients because if we don’t get feedback and receive them at the clinic, we must follow them in the community so that we continue with the process so that transport refund is not there. We also need airtime to remind them so at times we have that challenge.”*

## Discussion

This study aimed to explore the HCWs’ perspectives regarding barriers and facilitators to pediatric HIV status disclosure, with a focus on guideline utilization in eastern Uganda. The findings indicated that barriers affecting the capability, opportunity, and motivation of HCWs to support disclosure and utilization of guidelines were at individual, interpersonal, institutional, community, and policy levels.

Barriers related to capability included lack of pediatric HIV disclosure training (institutional level) while those pertaining to opportunity were limited access to guidelines (policy level), unstable home environment (community level), and HIV-related stigma (individual, interpersonal, and community levels). Barriers related to the motivation of HCWs included limited health funding (policy level) lack of preparatory procedures (institutional level) and delayed disclosure (individual level). Facilitators were mainly at individual, interpersonal, and institutional levels. HCW’s awareness of responsibilities was a facilitator related to capability (individual level), while opportunity-related facilitators included peer support, teamwork (interpersonal level), and orphanhood status (individual level). Facilitators related to motivation included emotional reward to HCWs (individual level), monitoring, checklists, and supervision (institutional level).

These findings highlight important gaps at different levels that need to be addressed to improve HIV disclosure among CALH. For example at the institutional level, this study highlights the need for further training of healthcare workers regarding pediatric HIV disclosure and the use of existing guidelines to bridge the knowledge gap and enhance their capability to support the disclosure process. WHO noted that one of the commonest barriers to HIV status disclosure among CALH was a lack of knowledge about the disclosure process among healthcare workers [25]. In this study, the lack of knowledge was mainly concerning disclosure guidelines and processes as well as the benefits of disclosure. Training HCWs does not only improve knowledge but also skills for disclosure which can motivate and build confidence among healthcare providers [18]. Additionally, HCWs should be encouraged to incorporate disclosure into their routine activities and plan for it ahead of time as it not only facilitates disclosure but also the use of guidelines during the disclosure process. Adequate training of HCWs, coupled with disclosure preparedness at the health facility level by putting in place well-streamlined preparatory procedures for HIV disclosure and ensuring proper access to all relevant guidelines can improve capability, provide a conducive environment, and also motivate staff to be involved in disclosure-related activities. Additionally, awareness of responsibilities also enhances healthcare workers’ psychological ability to engage in disclosure activities, pointing to the need to regularly sensitize or remind staff about their job descriptions and respective roles in the HIV disclosure process.

Also, some of the highlighted barriers to HIV status disclosure related to the social and physical environments for disclosure align with existing literature and hence need to be addressed. These include HIV-related stigma (all types) which has been documented severally, as a major social hindrance to disclosure among all groups of people living with HIV [26–29]. The most common and debilitating types are self/internalized and anticipated stigma experienced both by caregivers of CALH which make people with HIV feel unworthy or think that they will be discriminated against by others in their societies [28,30]. Whereas internalized stigma is characterized by feelings of shame, guilt, and unworthiness, anticipated stigma is marked by caregivers’ fear of negative emotional reactions such as anger, excessive sadness, and suicidal behavior from the child or rejection and maltreatment by the partner especially for those couples who have never disclosed their status to one another. This is in line with several studies that have highlighted caregivers’ fear of negative emotional reactions from partners, family, and close relatives as a major hindrance to HIV status disclosure to children [16,31,32]. This study further highlights that these reactions can equally affect HCWs’ willingness to facilitate disclosure if directed to them. Enacted stigma can also limit disclosure of HIV status because when people become discriminated against, they lose motivation to disclose to their children for fear that they will face the same [27,30]. In communities where HIV has been linked to immorality or promiscuity, it becomes difficult for parents to disclose since they fear that if the community gets to know their status, they will be judged and discriminated against [33,34]. Additionally, in such a stigmatizing social environment, some HCWs may choose to dissociate themselves from children with a highly stigmatized condition and their caregiver which further complicates the disclosure process.

On the contrary, orphanhood status was found to make disclosure of HIV status to the child easier because it eliminates the stigmatized individual leaving the child with no one to blame hence making the social environment more disclosure-friendly. Among couples that have never disclosed to each other, orphanhood also eliminates the risk and psychosocial complications of unintended disclosure such as interpersonal conflicts hence making disclosure easier by other relatives [32].

Regarding the physical environment, this study also found that unstable homes characterized by unavailable, or unreliable caregivers and those who have not disclosed their status to other people limit HIV disclosure to children. This means that children (including orphans) with such caregivers may be at higher risk for non-disclosure hence they may need more support and psychoeducation to disclose to their children. This comes as a strong consideration for settings where there is an extended family system that raises the orphaned children with frequent changes of caregivers or those who are elderly or disabled that cannot easily sustain the disclosure process [35,36]. Hence a stigma-free environment with stable homes can provide a better opportunity for HCWs and caregivers of CALH to carry out disclosure.

The other important institutional factors in the social environment that created better opportunities for HCWs to support disclosure were teamwork and peer support. Teamwork was found to be a strong facilitator of disclosure because it improves group dynamics among HCWs where each member plays their role in the disclosure process hence making work easier and less burdensome to a single individual. This underscores the need for a multidisciplinary approach to comprehensive HIV care for good healthcare outcomes [37,38]. Also, peer support among HCWs plays a very vital role in pediatric HIV status disclosure by promoting the utilization of guidelines through peer learning. Additionally, existing peer support systems for CALH (such as YAPS) were reported as a strong block to disclosure. these YAPS share their experiences and provide additional information about HIV/AIDS to other children and adolescents which helps them to cope better during and after disclosure. The HCWs also highlighted that strategies geared towards enhancing caregiver-caregiver support through disclosure can improve disclosure as those who have successfully disclosed can share their experiences with those struggling to undergo the process. In some cases, supported disclosure can be used as a form of assisted disclosure if the caregiver feels not confident enough to do it alone [39,40]. These forms of peer or social support align with WHO recommendation of assisted disclosure strategies such as “caregiver buddy” and others which can be utilized to improve disclosure [40]. In countries like Uganda where professional staffs are limited, existing peer and social support systems such as YAPS, linkage facilitators, expert clients, and other community health workers can be explored as alternatives that can cover the existing gap as these have been found to work for other populations living with HIV [41–43]

Regarding motivation, the study highlighted that delayed disclosure was an important barrier to utilization of guidelines as it creates a sense of urgency which increases the risks of unsafe disclosure and makes following disclosure guidelines difficult. This could also be worsened by fear of negative emotional reactions from such older children hence continuously demotivating HCWs and caregivers to those children from disclosing. That notwithstanding, HCWs reiterated the need to prepare caregivers to initiate the disclosure process early enough as per the recommended (pre-school stage) guideline to mitigate such shortfalls [13]. However, several studies have also reported that some caregivers fear disclosing to children because of age where they fear that young children may not comprehend the concepts related to HIV or cannot keep a secret [17,31,44]. Hence, whereas guidelines recommend that disclosure should be done according to the child’s maturity, age, and cognitive abilities, this seems to pose a practical challenge of assessing the acceptable level of maturity and readiness by HCWs or caregivers to initiate disclosure leading to delays.

Among HCWs, emotional reward was a strong automatic motivator to facilitate disclosure, and this was mainly achieved by being appreciated by caregivers, CALH, or their employers/superiors for accomplishing such a difficult task. The appreciation gives a sense of satisfaction at work hence providing both intrinsic and extrinsic motivation for sustained behavioral change [45–48]. Also being aware that HIV status disclosure is their responsibility and part of the job description could motivate HCWs to engage in it as an extrinsic motivation since it gets them paid. Furthermore, the motivation of HCWs can be enhanced by reinforcing the observation of the various checklists that have been found to encourage HCWs to initiate the disclosure process and continue supporting caregivers to disclose. Some of the checklists create a state of urgency for disclosure to avoid poor clinical outcomes that have been linked to non-disclosure among children [49,50]. This along with routine monitoring and supervision of HCWs’ disclosure activities can improve the utilization of disclosure guidelines. However, it is important to note that some of the checklist considerations like children’s poor clinical status that create a state of urgency may make prompt following of disclosure guidelines by HCWs difficult.

Lastly, at the policy level, inadequate health funding remains a major challenge to HIV status disclosure among children as it leads to inadequate staffing which creates work overload among HCWs and time constraints due to competing interests. Specifically, the resultant time constraints limit the use of existing guidelines because it would make the process lengthier. This is in line with the existing literature and guidelines which highlight that disclosure is a process rather than an event [13,39,51]. Whereas limited health funding has often been highlighted as a challenge in the health sectors of most developing countries, there is a dire need for health policymakers to revise their funding priorities to ensure good health service provision, especially to such vulnerable populations [18,52–54]. In addition to improved staffing, adequate funding can ensure the availability of disclosure guidelines in clinics, and improve facilitation and monitoring/supervision of all disclosure-related activities including prompt following of guidelines during the disclosure process and encouraging teamwork among staff. On the contrary, limited health funding affects the disclosure environment by not providing adequate spaces to ensure privacy and confidentiality during the disclosure process which is a vital requirement [55]. Generally, adequate health funding is essential for improving the capability of HCWs through providing disclosure training, ensuring a conducive social and physical environment, and motivating HCWs to support disclosure and utilize guidelines.

### Study limitations

This was a facility-based study that assessed barriers and facilitators to pediatric HIV status disclosure considering only HCWs’ perspective, hence there is a need to consider other stakeholders in the disclosure process such as caregivers and policymakers. Additionally, there is a possibility of social desirability bias in some of the responses from study participants especially about issues concerning their work and workplace.

## Conclusions

As HIV status disclosure remains a significant public health challenge in resource-limited settings, several modifiable factors can be addressed to minimize the magnitude and effects of non-disclosure and improve the utilization of guidelines in the disclosure process. Therefore, interventions are needed to address these factors which involve multiple stakeholders from individual to national/policy level using a multi-pronged and multidisciplinary approach with rigorous stakeholder engagement. These may focus mainly on training HCWs and ensuring that they are aware of their roles in the disclosure process to boost their capability. Also providing a favorable home, community, and health facility environment that is stigma-free, encourages teamwork and peer support with access to existing guidelines can encourage HIV disclosure. At a national level, there is a need for adequate health funding to improve staffing levels and reduce work overload, while motivating HCWs at institutional and individual levels can also improve disclosure and utilization of guidelines. Hence there is a need to develop implementation strategies that address the above gaps to encourage the utilization of these guidelines and hence improve disclosure among children.

## Supporting information

S1 Table COnsolidated criteria for REporting Qualitative research (COREQ) checklist. (DOC)

S1 Fig Summary of themes for barriers and facilitators to pediatric HIV status disclosure categorized using the social-ecological model. (TIF)

S2 Table Interview guide for healthcare workers. (DOC)
