# Supplementary material for: Healthcare workers’ perspective about barriers and facilitators to pediatric HIV status disclosure in eastern Uganda using capability opportunity and motivation of behavior change model
Source: PLOS Glob Public Health. 2025 May 29;5(5):e0004662. doi: 10.1371/journal.pgph.0004662 (PMC12121738; doi:10.1371/journal.pgph.0004662)
Supplement: S2 Table — (DOC) [file pgph.0004662.s003.doc]

Interview guide for healthcare worker’s perspectives regarding barriers and facilitators for HIV status disclosure.

| **Domain** | **Constructs** | **Interview question** |
| --- | --- | --- |
| Capability | Psychological ability | Do you ever facilitate HIV status disclosure among children?  Do you know about the WHO or MoH guidelines for HIV status disclosure to children? If yes, have you looked through them?  What do you think the guidelines say about HIV status disclosure to children?  Is HIV status disclosure to children something you usually do (Would you remember)?  What do you think about using the guideline during disclosure process? What do you think about it as part of your role?  Would you remember to follow the guidelines when facilitating HIV status disclosure? How would that happen? |
|  | Physical ability | How do you disclose HIV status to children? Have you ever used any guideline?  If yes, which ones? |
| Opportunity | Physical environment | To what extent does the surrounding environment facilitate or hinder HIV status disclosure?   - Space from where to facilitate caregiver to disclose to child - Competing tasks or time constraints - Access to the WHO or MoH disclosure guidelines |
|  | Social environment | To what extent do social influences facilitate or hinder facilitation of HIV status disclosure? (prompt for peers, seniors, patients, relatives)   - See others facilitate disclosure - See others using the guidelines when facilitating disclosure - Support supervision when facilitating disclosure |
| Motivation | Reflective mechanism | What do you think about HIV status disclosure process?  Do you think you need to use the guideline during disclosure process? Why? How easy or difficult do you think it is to facilitate disclosure process?  How easy or difficult do you think it is to follow the guideline during disclosure process?  What challenges do you think are there in disclosing HIV status? What support would you need to facilitate disclosure?  Do you think you would need guidelines to facilitate? Why?  How confident are you that you can use the guidelines to facilitate disclosure once they are provided?  What would motivate you to facilitate disclosure? |
|  | Automatic mechanism | Does facilitate HIV status disclosure process cause some emotions in you?   - If so, what? - To what extent do emotional factors facilitate or hinder you from facilitating disclosure?   What preparatory or introductory steps are there at health facility level to help you during disclosure process? Probes for   - Preparation on how to facilitate disclosure (with or without) the guideline - Measures (regulations) of encouraging you to facilitate disclosure |
